# Coenzyme Q10 restores oocyte mitochondrial function and fertility during reproductive aging

**DOI:** 10.1111/acel.12368

**Published:** 2015-06-26

**Authors:** Assaf Ben-Meir, Eliezer Burstein, Aluet Borrego-Alvarez, Jasmine Chong, Ellen Wong, Tetyana Yavorska, Taline Naranian, Maggie Chi, Ying Wang, Yaakov Bentov, Jennifer Alexis, James Meriano, Hoon-Ki Sung, David L Gasser, Kelle H Moley, Siegfried Hekimi, Robert F Casper, Andrea Jurisicova

**Affiliations:** 1 Lunenfeld-Tanenbaum Research Institute, Mount Sinai Hospital25 Orde Street, Toronto, ON, M5T 3H7, Canada; 2 TCART Fertility Partners150 Bloor W, Toronto, ON, M5S 2X9, Canada; 3 Department of Physiology, University of Toronto1 King’s College Circle, Toronto, ON, M5S 1A8, Canada; 4 Department of Obstetrics and Gynecology, Washington University in St. Louis660 S. Euclid Avenue, St. Louis, MO, 63110, USA; 5 Department of Biology, McGill University3649 Promenade Sir William Osler, Montreal, QC, H3G 0B1, Canada; 6 Department of Obstetrics and Gynecology, University of Toronto92 College Street, Toronto, ON, M5G 1L4, Canada; 7 LifeQuest Centre for Reproductive Medicine655 Bay St, Toronto, ON, M5G 2K4, Canada; 8 Department of Genetics, University of Pennsylvania575 Clinical Research Building, 415 Curie Boulevard, Philadelphia, PA, 19104-6145, USA

**Keywords:** Mitochondria, mouse models, molecular biology of aging, individual, fecundity, anti-aging

## Abstract

Female reproductive capacity declines dramatically in the fourth decade of life as a result of an age-related decrease in oocyte quality and quantity. The primary causes of reproductive aging and the molecular factors responsible for decreased oocyte quality remain elusive. Here, we show that aging of the female germ line is accompanied by mitochondrial dysfunction associated with decreased oxidative phosphorylation and reduced Adenosine tri-phosphate (ATP) level. Diminished expression of the enzymes responsible for CoQ production, *Pdss2* and *Coq6,* was observed in oocytes of older females in both mouse and human. The age-related decline in oocyte quality and quantity could be reversed by the administration of CoQ10. Oocyte-specific disruption of *Pdss2* recapitulated many of the mitochondrial and reproductive phenotypes observed in the old females including reduced ATP production and increased meiotic spindle abnormalities, resulting in infertility. Ovarian reserve in the oocyte-specific *Pdss2*-deficient animals was diminished, leading to premature ovarian failure which could be prevented by maternal dietary administration of CoQ10. We conclude that impaired mitochondrial performance created by suboptimal CoQ10 availability can drive age-associated oocyte deficits causing infertility.

## Introduction

Female fertility is one of the first physiological functions adversely affected by aging. Female fecundity starts declining at age 32 and decreases more rapidly after age 37 (O’Connor *et al*., 1998). This decrease in the probability of conception occurs in spite of continuing ovulatory cycles (te Velde & Pearson, 2002). Although neuroendocrine and uterine factors contribute to the age-related decline of successful pregnancy, the consistent live-birth rate of pregnancies from oocyte donation in aging women suggests that the decline in oocyte quality is the major contributing factor responsible for infertility with aging. Maternal aging is known to trigger a series of molecular alterations that drive the defects in chromatid separation (Chiang *et al*., 2011) and chromosome decondensation, as well as spindle detachment causing chromosomal misalignment (Battaglia *et al*., 1996; Liu & Keefe, 2002). However, only a few targets responsible for these changes have been identified, and no treatment thus far has been successful in improving the chances of a live birth in women of advanced maternal age.

Decline in female reproductive capacity with aging is accompanied by depletion of ovarian reserve. In human, progressive loss of ovarian follicles is nonlinear and becomes accelerated with age, especially after 38 years of age (Faddy, 2000). Molecular pathways behind increased loss of germ cells in aged ovaries are poorly understood. Increased DNA damage due to less active DNA repair machinery is one possible trigger for oocyte loss (Titus *et al*., 2013). The complex process of oocyte maturation prior to ovulation involves nuclear, cytoplasmic, and epigenetic changes culminating with the formation of the meiotic spindle. All of these processes require energy, which is provided by mitochondria mostly via oxidative phosphorylation – OXPHOS (Dumollard *et al*., 2007), as the alternative energetic process of glycolysis in the oocyte is limited due to low expression of phosphofructokinase (Leese & Barton, 1984). The bioenergetic state of the oocyte influences its developmental competence with correlations between implantation potential, ATP content (Van Blerkom *et al*., 1995), and mitochondrial membrane potential (Wilding *et al*., 2001). Furthermore, interference with OXPHOS or with mitochondrial function leads to arrest of oocyte maturation, chromosomal misalignment, and compromised embryo development (Takeuchi *et al*., 2005; Thouas *et al*., 2006; Wyman *et al*., 2008).

ATP production via OXPHOS involves the action of the electron transfer chain consisting of five complexes located on the inner mitochondrial membrane. Complexes I and II oxidize products of the tricarboxylic acid (TCA cycle) and transfer the electrons to ubiquinone, also known as coenzyme Q (CoQ). The electrons are transferred to complexes III and IV, creating a proton gradient which culminates in the generation of ATP by complex V. CoQ is pleiomorphic having critical antioxidant properties, controlling cellular redox, altering various signaling pathways, and influencing transcriptional activity of cells and is required for the activity of succinate dehydrogenase (Crane, 2001; Quinzii *et al*., 2010).

Several observational studies demonstrated a tissue-specific decline in CoQ levels with age (Kalen *et al*., 1989; Miles *et al*., 2004). *De novo* CoQ production involves a complex but poorly understood biochemical pathway, depending on the activity of at least 10 different enzymes. The benzoquinone ring of CoQ is synthesized from the amino acid tyrosine or phenylalanine, and the tail is produced from acetyl-CoA by the mevalonate pathway through the action of tetrameric decaprenyl diphosphate synthase, encoded by the *Pdss1* and *Pdss2* genes. Upon condensation of the ring with the polyprenyl tail by the COQ2 enzyme, the ring structure is modified by decarboxylation, hydroxylation, and methylation, mediated by enzymes encoded by *Coq 3, 6, and 7*. CoQ proteins form a large mitochondrial complex (Tran & Clarke, 2007), and the presence of all protein components is required to maintain its stability (Wang & Hekimi, 2013). The aim of this study was to determine whether impaired oocyte mitochondrial function could be improved by supplementation with mitochondrial energy-production stimulants and to determine whether oocyte-specific inhibition of *CoQ* synthesis could recapitulate the reproductive abnormalities seen with aging.

## Results

**Fig 1:**
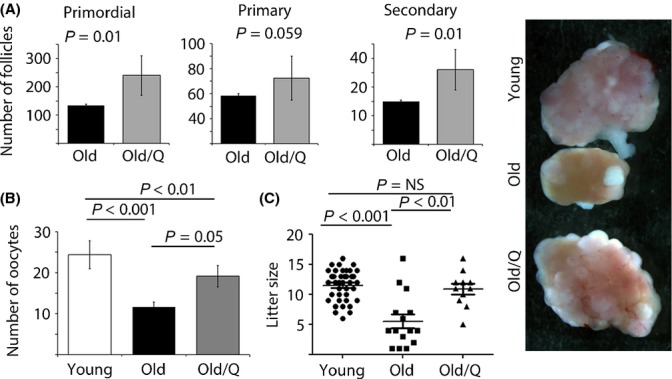
Impact of CoQ10 treatment on ovarian reserve and breeding performance in an aged mouse model. (A) Ovarian reserve was significantly higher in old vehicle mice treated with CoQ10 for a period of 15 weeks (*n* = 9/age and treatment) evidenced by significantly higher number of resting primordial and growing secondary follicles. Values represent average follicle numbers ± SEM. Images of stimulated ovaries from each group are shown on the right – magnification 50×. (B) Number of ovulated oocytes collected after hormonal stimulation of young (*n* = 20), old vehicle-treated (*n* = 16), and old CoQ10-treated dams (*n* = 16). Values represent average number of oocytes per female ± SEM. (C) Litter size in young (*n* = 39), old vehicle-treated (*n* = 15), and old CoQ10-treated mice (*n* = 11). The number of live pups born to dams in the 13th month of age was decreased, but normalized after CoQ10 supplementation. Each female produced only one litter during breeding trial. Scatter plot data are shown as mean per female ± SEM.

As maternal aging is accompanied by reduction of mitochondrial function in oocytes (Kujjo *et al*., 2013), we treated aged dams with known stimulators of mitochondrial bioenergetics, CoQ10, alpha lipoic acid, and resveratrol. CoQ10 significantly improved ovulation rates in this model (Fig. S1A). Thus, we focused our further experiments to determine whether CoQ10 can ameliorate other aging-mediated phenotypes in the female germ line. After confirming that ovarian response was improved in a larger cohort of females treated with CoQ10 (Fig.1), we evaluated whether ovarian reserve and breeding performance with aging can also be improved. While substantial ovarian reserve was lost during a 3-month treatment (from 9 to 12 months Fig. S1B), ovarian histomorphometry confirmed increased numbers of primordial, preantral, and antral follicles in the CoQ10-treated group compared to the aged-matched vehicle-treated cohort (Fig.1A). As preservation of ovarian reserve does not necessarily mean production of better quality oocytes, we subjected females to a breeding trial. The reduced litter size observed in the old vehicle-treated dams was normalized with CoQ10 (Fig.1C). Thus, CoQ10 supplementation not only preserved the ovarian follicle pool, but also facilitated ovulation of gametes able to support normal development.

**Fig 2:**
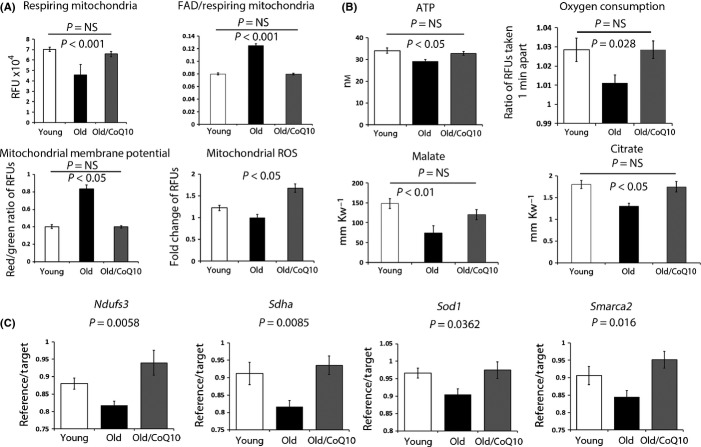
Improvement in mitochondrial function in oocytes of old vehicle mice after CoQ10 supplementation. (A) Oocytes from young, old vehicle and old CoQ10 mice were stained with MitoTracker Red, JC-1, and Mitosox or examined for green autofluorescence (FAD). The respiring mitochondrial pool (MitoTracker Red) was reduced in oocytes from old vehicle mice compared to young or old CoQ10, which were not different from each other. The ratio of oxidized FAD (FAD^++^)/MitoTracker Red increased in old oocytes. The mitochondrial membrane potential (MMP) increased in old oocytes, while ROS production (Mitosox) decreased. These aging effects were normalized by CoQ10 treatment. Values represent random fluorescence units (RFUs) per oocyte ± SEM. For all experiments, individual oocytes were used and groups contained *n* = 15–25 oocytes/age/treatment. (B) ATP (nm) and TCA cycle metabolites (millimole of substrate per kilogram wet weight per oocyte) were evaluated in individual oocytes from young (*n* = 7–15), old vehicle (*n* = 8–14), and old CoQ10 (*n* = 11–17). Oxygen consumption is expressed as a ratio of fluorescent signals obtained by a scan 1 min apart and reflects oxidative decay of the probe per oocyte. Data shown are mean ± SEM. (C) Expression levels of genes involved in mitochondrial function *(Ndufs3, Sdha, and Sod1*) and chromatin organization *(Smarca2)* in ovulated oocytes were reduced with aging and improved after treatment with CoQ10 (mean ± SEM). Each sample contained a pool of 3 oocytes (*n* = 4 young, *n* = 6 old vehicle and *n* = 5 old CoQ10 pools), and data are shown as the ratio of reference (actin)/target (studied) transcript.

Mitochondrial dysfunction has been implicated in oocyte aging (Bentov *et al*., 2011). As CoQ is a well-known component of the electron transport chain, we next evaluated whether CoQ10 treatment could improve mitochondrial performance in oocytes. We determined that the pool of respiring mitochondria was decreased in aged oocytes and increased by CoQ10 treatment to the level similar to that of young controls (Fig.2A). Decreased mitochondrial activity in aged oocytes was reflected by lesser reduction of FAD++ to FADH2 as the oxidized (fluorescent) FAD++ to MitoTracker (active mitochondrial pool) ratio was increased with age and this was normalized by CoQ10 (Fig.2A). On the contrary, mitochondrial membrane potential was elevated with aging (Fig.2A) and restored to levels seen in the young animals after CoQ10 exposure. During completion of meiosis I, mitochondrial output and ATP demands are dramatically increased (Dalton *et al*., 2014). This can be monitored by increase in mitochondrially derived reactive oxygen species (ROS – Mitosox). ROS levels decreased with aging and were restored in the oocytes of CoQ10-treated dams (Fig.2A). Both ATP output and oxygen consumption were decreased with aging and significantly increased upon CoQ10 administration (Fig.2B). Consistent with these outcomes, metabolites of the TCA cycle, including citrate, malate, and to a lesser extend fumarate were reduced with aging and increased with CoQ10 treatment to the levels seen in the young controls (Figs2B and S2). Outcomes of these studies indicate the inhibition of the TCA cycle activity is a major reason for poor mitochondrial function in aged oocytes.

Using previously published outcomes of microarrays studies on aged murine oocytes (Hamatani *et al*., 2004; Pan *et al*., 2008), we established that expression of genes involved in mitochondrial metabolism was reduced by aging. Expression of *Sdha* and *Nduf3*, the mitochondrial ROS scavenger *Sod1* as well as the ATP-dependent chromatin regulator *Smarca2* (Fig.2C) were all significantly decreased in the oocytes of the aged animals and increased in the CoQ10-treated aged group. In contrast, CoQ10 supplementation had no significant impact on expression of genes involved in transcription or chromatin organization *(Cggbp1, Arf1, Ezh2, Bmi1, Rbbp4, Kpna2*) (Fig. S2). Thus, we conclude that maternal treatment with CoQ10 boosts mitochondrial function in aged oocytes, restores activity of TCA cycle, and normalizes energy production.

**Fig 3:**
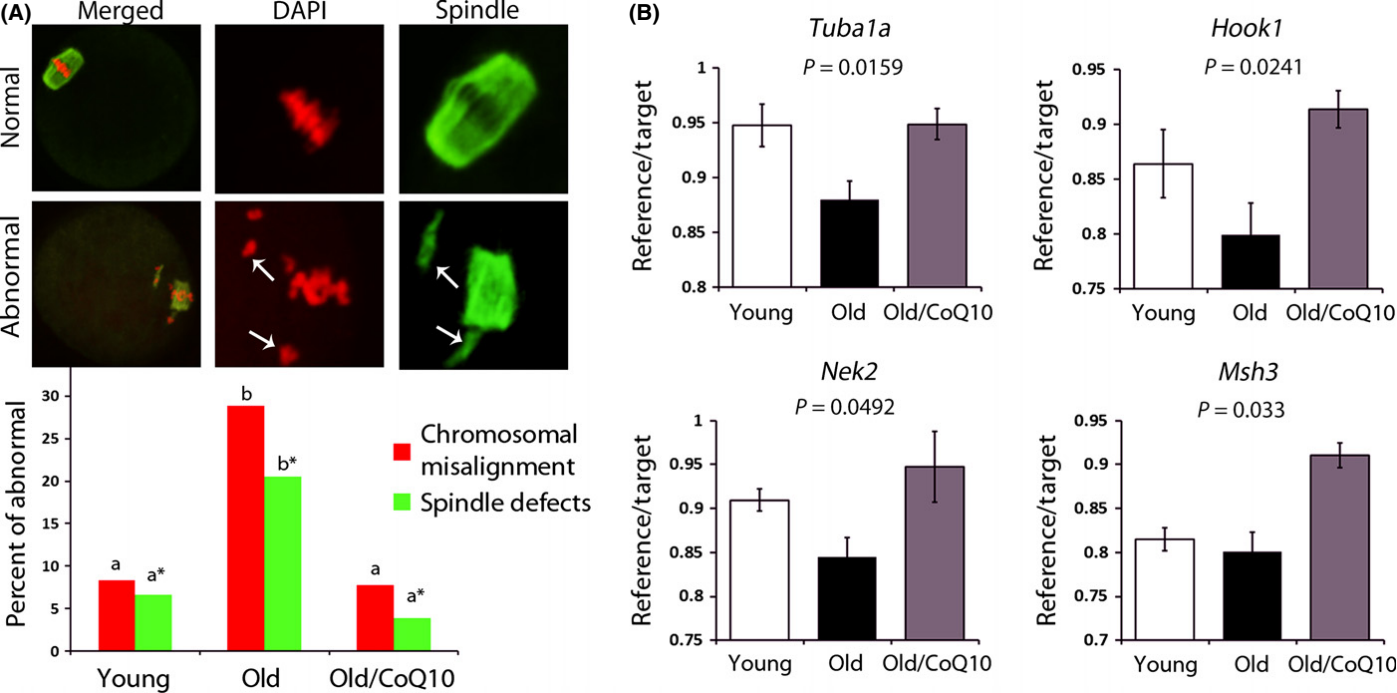
CoQ10 rescues spindle defects in aging oocytes. (A) Percent of chromosomal or spindle misalignment in ovulated oocytes from young (*n* = 60), old (*n* = 73), and old CoQ10 (*n* = 51) mice. Oocytes were stained with antitubulin antibody (green) and DAPI (red). Representative images of normal spindle (barrel shaped) and chromosome alignment (toothbrush appearance) were considered normal. Arrows demonstrate detachment of chromosomes from spindle or misshaped spindle organization. Letters (a vs. b and a* vs. b*) are significantly different from each other (*P* < 0.05). (B) Expression of genes implicated in spindle formation/attachment and meiotic execution *(Tuba1a, Hook1, Nek2, and Smarca2)* in oocytes was reduced with aging and improved after treatment with CoQ10. Each sample contained a pool of 3 oocytes, and each age category was represented by *n* = 4 young, *n* = 6 old, and *n* = 5 old CoQ10 pools, and data are shown as the mean ratio of reference (actin)/target ± SEM.

Various mouse models have linked bioenergetic status of oocytes to abnormal meiotic outcomes, particularly defects in spindle positioning and chromosome scattering (Wang *et al*., 2009; Luzzo *et al*., 2012). We thus assessed the metaphase II spindle and chromosomal alignment of ovulated oocytes. As previously reported, aging was accompanied by the production of oocytes with higher rates of spindle defects and chromosomal misalignment. CoQ10 treatment in the old mice restored normal spindle appearance and prevented chromosomal scattering to a degree indistinguishable from young dams (Fig.3A). In addition, transcript levels of genes involved in meiotic progression such as *Tuba1a, Nek2,* and *Hook1* were all significantly increased after CoQ10 treatment in the old animals (Fig.3B), while no change was observed for *Ccna2* (Fig. S2). Most importantly, the litter sizes of live-born pups in the CoQ10-treated older mice were similar to the litter sizes seen in the young animals (Fig.1C).

The source of CoQ10 in most tissues is believed to originate from endogenous production because the bioavailability of CoQ10 from the diet is very low. In rodents, the major CoQ variant is CoQ9 with a variable content of CoQ10 that differs among tissues. Similar to liver, the ovary appears to efficiently uptake CoQ10 from external sources without dramatic impact on endogenous CoQ9 levels. (Table S1).

**Fig 4:**
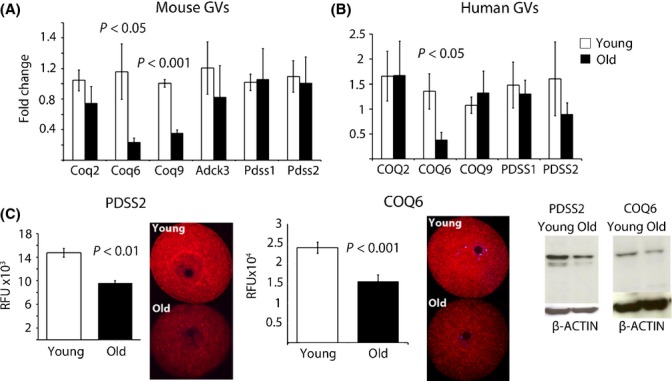
Reduced expression of CoQ10 biosynthesis genes in oocytes with aging. (A) Fold change in mRNA level of pooled GVs (3 per sample; *n* = 6 young, *n* = 6 old) were normalized to young age. Transcripts encoding enzymes *Pdss1 and Pdss2* did not change with aging, but the expression of the enzymes involved in modifying the hydroxybenzoate ring (*Coq6* and *Coq9)* significantly decreased. (B) Fold change in mRNA level of CoQ10 synthesis genes in single human GV oocyte per patient (*n* = 8 patients <32 years old, *n* = 8 patients >39 years old females). Data are shown as mean ± SEM and are normalized to actin. (C) Immunocytochemistry of GV oocytes exposed to anti-PDSS2 and anti-COQ6 antibodies from young (*n* = 9) and old vehicle mice (*n* = 5). Values represent mean fluorescence units ± SEM. Western Blot of whole ovarian lysates from young (3 months old) and aged mice (12 months old) blotted with anti-PDSS2, anti-COQ6, and anti-actin antibodies.

We next proceeded to determine whether aging oocytes express altered levels of enzymes involved in CoQ biosynthesis. Quantitative RT–PCR of growing (GV) stage oocytes revealed a significant decrease in *Coq6* and *Coq9* expressions (Fig.4A) but no change in the *Pdss1/2* transcript levels. To establish whether human oocytes demonstrate the same molecular changes as the mouse, we assessed expression of these targets in GV oocytes obtained from patients undergoing IVF treatment. Similar to the mouse, a significant decline in *CoQ6* expression was observed in the oocytes of older patients (Fig.4B). Western blot analysis of ovaries isolated from young and old dams revealed reduction in both PDSS2 and CoQ6 proteins (Fig.4C). Immunolocalization on ovarian sections confirmed expression of PDSS2 and CoQ6 in oocytes of growing follicles (from the secondary follicle stage onwards), as well as lower, albeit detectable, expression in the stroma, theca, cumulus, and luteinized granulosa cells (Fig. S4). As declining levels of PDSS2 and CoQ6 in ovarian lysates could reflect altered tissue composition (e.g., decreased follicle pool), we performed immunocytochemistry on GV stage oocytes isolated from young and old females. Both PDSS2 and CoQ6 were significantly reduced with age (Fig.4C) in oocytes, although we did not observe significant reduction in CoQ9/10 level in total ovarian lysates in aged animals (Table S1). As oocytes only represent a very small fraction of the total ovarian tissue content, changes that occur in these cells would be untraceable.

**Fig 5:**
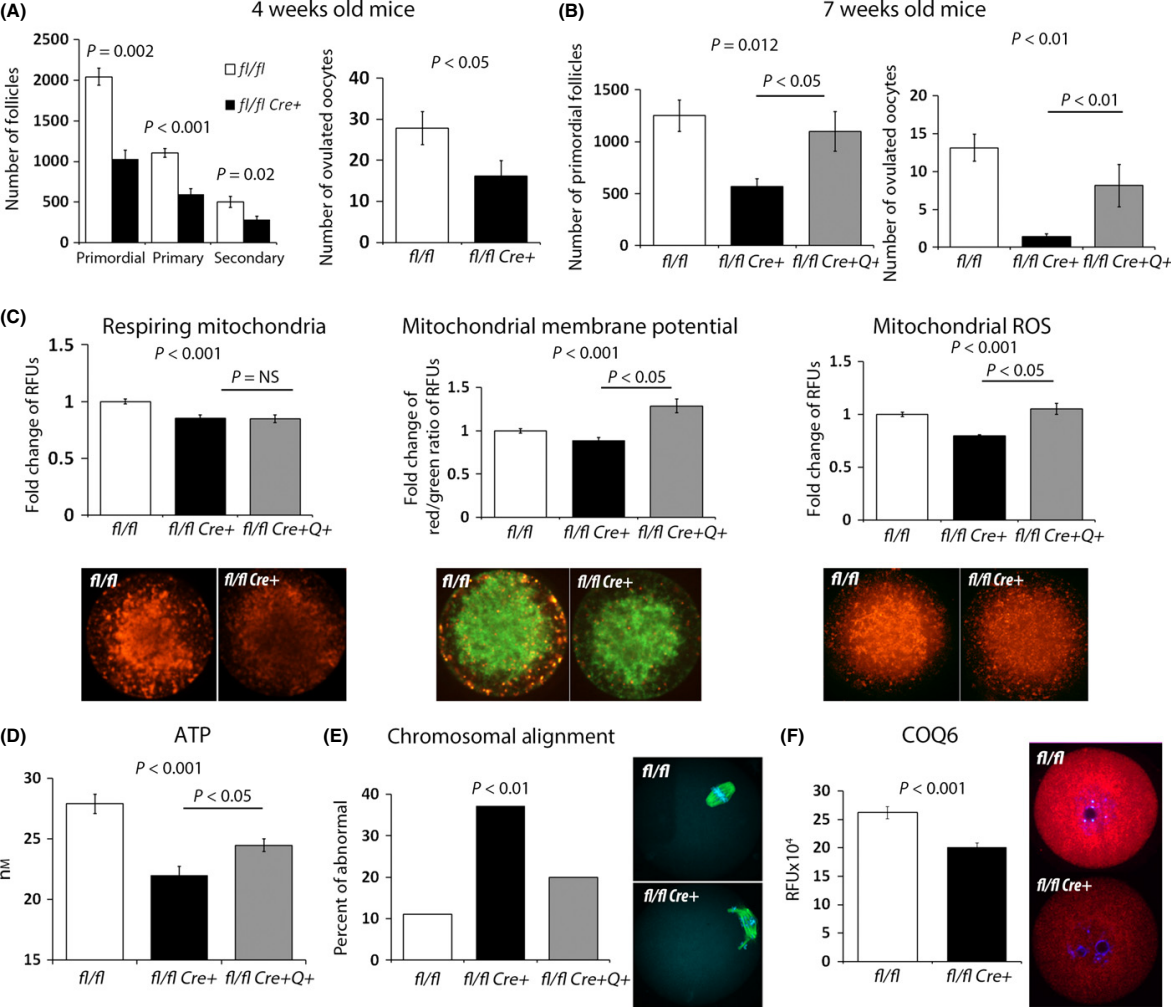
Decreased ovarian reserve and oocyte quality in *Pdss2 *^*fl/fl Cre+*^ mice is rescued by CoQ10 supplementation. (A) The follicular count of ovaries (mean ± SEM) from 4-week-old vehicle mice revealed decreased ovarian reserve in *Pdss2 *^*fl/fl Cre+*^ (*n* = 8) compared to *Pdss2* ^*fl/fl Cre−*^ mice (*n* = 5). Follicle loss is reflected also by the reduced ovulation rate (*n* = 8 *Pdss2 *^*fl/fl Cre+*^, *n* = 16 *Pdss2* ^*fl/fl Cre−*^). (B) Treatment with CoQ10 from birth till 7 weeks of age prevented loss of ovarian reserve (mean ± SEM) and improved the ovulation rate triggered by *Pdss2* deficiency. (C) Oocytes from *Pdss2 *^*fl/fl Cre+*^ exhibit mitochondrial dysfunction with decreased respiring mitochondrial pool, reduced mitochondrial membrane potential (MMP), and decreased ROS production. Similar to aging, MMP and mitochondrial ROS levels were significantly improved under CoQ10 supplementation. (D) ATP output per oocyte improved by CoQ10 administration in *Pdss2 *^*fl/fl Cre+*^ females. All data shown are mean ± SEM obtained from 20–40 oocytes. (E) Chromosomal misalignment was significantly more frequent in oocytes from *Pdss2 *^*fl/fl Cre+*^ (*n* = 62) compared to *Pdss2* ^*fl/fl Cre−*^ (*n* = 118), and this was corrected by CoQ10 administration (*n* = 56). (F) Anti-COQ6 protein level in growing GV oocytes. *Pdss2 *^*fl/fl Cre+*^ oocytes present with significantly reduced levels of CoQ6 protein (*n* = 31 *Pdss2* ^*fl/fl Cre−*^, *n* = 44 *Pdss2 *^*fl/fl Cre+*^).

Previous work has established that disruption of *Pdss2* and prevention of CoQ synthesis are embryonic lethal. However, it is currently unknown whether the function of this enzyme is required for oogenesis. We used conditional disruption of *Pdss2* in oocytes to investigate whether interruption of CoQ10 synthesis will affect ovarian reserve. The ZP3-Cre^*3Mrt*^ line has a high efficiency of excision (∼98%), with activity restricted to oocytes initiated at the primordial follicle stage (Fig. S3A). PDSS2 levels were reduced in growing GV oocytes of *Pdss2 *^*fl/fl Cre+*^ mice (Fig. S3B), and immunoreactivity was virtually absent in fully grown oocytes (Fig. S3C). Although the *Pdss2 *^*fl/fl Cre−*^ females had an average of 6 pups per litter, the *Pdss2 *^*fl/fl Cre+*^ females (*n* = 4) did not produce any viable offspring during an entire 3-month breeding trial when crossed with males of proven fertility (Fig. S3D). By 4 months of age, *Pdss2 *^*fl/fl Cre+*^ female ovaries contained no healthy follicles and were accompanied by a hypotrophic uterus, a condition resembling premature ovarian failure. The ovulation rate at 4 weeks of age, shortly after the onset of puberty, revealed a decreased number of ovulated oocytes, and the ovarian reserve of the *Pdss2 *^*fl/fl Cre+*^ mice was already severely compromised, evidenced by the loss of 50% of follicles (Fig.5A,B).

We next investigated whether oocytes ovulated by young *Pdss2 *^*fl/fl Cre+*^ females exhibited mitochondrial phenotypes similar to those found in the old females. The respiring mitochondrial pool (e.g., MitoTracker Red), mitochondrial ROS, and ATP were all significantly decreased (Fig.5C,D). In addition, a higher proportion of oocytes from *Pdss2 *^*fl/fl Cre+*^ females contained misalignment of chromosomes, suggesting that disruption of the CoQ10 biosynthetic pathway could contribute to abnormal meiotic outcomes observed with increased maternal age (Fig.5E). As we have observed reduced CoQ6 levels in aged oocytes, we examined whether this outcome could be a response to PDSS2 deficiency. Indeed, *Pdss2-*deficient GV oocytes also expressed a reduced level of CoQ6 protein (Fig.5F).

To determine whether CoQ10 is the factor responsible for poor ovarian reserve caused by *Pdss2* deficiency, we exposed mothers during pregnancy/lactation to CoQ10 with subsequent dietary supplementation of pups with CoQ10 through suckling. At 2 months of age, control *Pdss2 *^*fl/fl Cre+*^ mice displayed severely reduced ovulation rates accompanied by reduction in ovarian reserve. This phenotype in the pups was partially corrected by lactational exposure to CoQ10 (Fig.5B). In addition, mitochondrial membrane potential, ATP production, and superoxide levels were improved in the CoQ10-supplemented animals, without a change in the respiring mitochondrial pool (Fig.5B,C). We also observed an improvement in spindle and chromosomal alignment (Fig.5D). Interestingly, exposure of young *Pdss2 *^*fl/fl*^ females to CoQ10 did not alter ovarian reserve, breeding, ovulation rates, or mitochondrial function (Fig. S4). These results indicate that diminished synthesis of CoQ created by oocyte-specific *Pdss2* disruption is sufficient to alter ovarian function, suggesting that deficiency of CoQ may contribute to the accelerated oocyte loss and poor pregnancy outcomes seen with aging.

## Discussion

In this study, we demonstrate that CoQ10 supplementation in an aged animal model delayed depletion of ovarian reserve, restored oocyte mitochondrial gene expression, and improved mitochondrial activity. As a result, more oocytes were ovulated in aged mice, developmental potential of the oocytes was improved, and more pups were born. The conditional disruption of the *Pdss2* gene resulting in CoQ deficiency in oocytes recapitulated many of the phenotypic changes characteristic of oocyte mitochondrial dysfunction associated with reproductive aging. These changes could be reversed by feeding the animals CoQ10. CoQ10 supplementation had no impact on ovarian reserve or oocyte quality of young females, suggesting no beneficial reproductive effect of CoQ10 in animals in which mitochondrial function is intact.

The reproductive aging process in mammals includes progressive reduction in ovarian follicular reserve with decreased oocyte quality. Increased loss of ovarian follicles with aging had been confirmed both in rodent and in human ovaries (Faddy *et al*., 1983; Faddy, 2000). We detected a significantly higher number of primordial follicles after 12 weeks of treatment with CoQ10. When we performed follicle counts, we did not see any dying primordial oocytes in aged ovaries, based on morphology and chromatin condensation. Therefore, the most likely explanation for the observed increase in the number of follicles is due to decreased atresia. The second alternative possibility to increase primordial follicle reserve is a decrease in recruitment toward growth. However, as we see slightly more primary oocytes in the CoQ10 treatment group, this is an unlikely explanation.

Previously published microarray studies comparing old and young oocytes revealed altered expression of genes responsible for mitochondrial function, oxidative stress responses, chromosome alignment, and ubiquitination (Hamatani *et al*., 2004; Pan *et al*., 2008). Ultrastructural examination of cellular organelles in aging oocytes confirmed the defects in mitochondrial architecture (Kujjo *et al*., 2013). Based on the outcomes of our study, it is clear that defects in mitochondrial performance occur concomitantly with the decline in breeding performance, and we attribute these changes to insufficient production of CoQ by oocytes. DNA damage is another contributing factor to oocyte aging (Titus *et al*., 2013), and it remains to be determined whether CoQ9/10 deficiency may accelerate DNA damage. However, it is clear that disruption of *Pdss2* in oocytes is sufficient to trigger mitochondrial defects reminiscent of aging.

CoQ10 is essential for mitochondrial activity as point mutations in enzymes responsible for CoQ10 synthesis are characterized by phenotypes involving high energy-consuming tissues such as the central nervous system, skeletal muscle, and the kidney (Lopez *et al*., 2006; Peng *et al*., 2008; Heeringa *et al*., 2011), and these symptoms can be partially alleviated by the administration of CoQ10. Unlike most rodent tissues in which CoQ9 is the predominant form of ubiquinone, the ovaries produce relatively high levels of CoQ10 and are able to efficiently uptake CoQ10 from external sources. We observed transcriptional decrease in *CoQ6* in aged oocytes and concomitant decrease in PDSS2 protein level. Reciprocal relationship exists between these two enzymes, as disruption of *Pdss2* triggered decrease of CoQ6 protein. Similar changes were previously described for *CoQ9* and CoQ7 (Garcia-Corzo *et al*., 2013). It is possible that disruption of key elements in the CoQ pathway could result in altered stability of whole CoQ enzyme complex (Wang & Hekimi, 2013).

In human, CoQ10 concentrations decrease after 30 years of age in some tissues (Morre *et al*., 2003; Miles *et al*., 2004), and perhaps this contributes to the aging process. The timing of the age-related decline in CoQ10 availability seems to coincide with the decline in fertility and the increase in embryo aneuploidies. Indeed, correlation between low plasma CoQ10 levels and spontaneous abortions were previously reported (Noia *et al*., 1996). In addition, levels of CoQ10 in the follicular fluid correlate with oocyte maturation and embryo grade during *in vitro* fertilization (Turi *et al*., 2012). Our results suggest that the oocyte is the beneficial target of CoQ10 supplementation. However, it is also possible that granulosa/cumulus cells and/or the uterine environment may also benefit and thus contribute to the increased reproductive capacity of CoQ10-treated females.

Due to limitations of technology, we have been unable to measure CoQ9/10 levels in oocytes. However, we demonstrate that the expression of multiple CoQ synthesis enzymes decreases with aging in human and murine oocytes, and cell-specific disruption of the CoQ synthesis pathway in young animals impacts negatively on oocyte quality. Thus, the oocytes, similar to podocytes in the kidney (Peng *et al*., 2008), dopaminergic neurons in the substantia nigra (Ziegler *et al*., 2012), and glial cells/neuroblasts in cerebellum (Lu *et al*., 2012), appear to be exquisitely sensitive to decreased CoQ levels. As CoQ10 administration improved breeding outcomes, slowed down follicle loss, and improved oocyte mitochondrial energetics in the aged animal model, it is tempting to propose that dietary CoQ10 supplementation could have beneficial reproductive effects in women seeking to conceive at a later age. While our results in an animal model appear promising, there are tremendous differences between aging mice and women, not the least of which is the order of magnitude difference in life expectancy. In relative terms, the use of CoQ10 for 12–16 weeks in a mouse is equivalent to about a decade in human. In addition, it is unknown whether a mitochondrial nutrient such as CoQ10 alone could reverse the impact of decades of environmental exposure of oocytes in human. Additional large-scale studies on dosing, length of treatment, and clinical outcomes safety are necessary before the use of CoQ10 in a clinical setting.

## Experimental procedures

### Animals and treatment

Institute of Cancer Research (ICR) female mice were obtained from Harlan Laboratories Inc. (Mississauga, Canada). All mouse experiments were performed in accordance with the Canadian Council on Animal Care (CCAC) guidelines for Use of Animals in Research and Laboratory Animal Care under protocols approved by animal care committees at Mount Sinai Hospital or the Toronto Centre for Phenogenomics. All mice were housed with free access to food and water, and kept on a 12-h:12-h light/dark cycle. For aging experiments, only proven (retired) breeders were used, while young controls were virgin females (7–8 weeks old).

For conditional disruption of *Pdss2* in oocytes, mice carrying the loxP allele of *Pdss2*^*tm1.1Dalg*^ were crossed with transgenic *ZP3-Cre*^*3Mrt*^ animals (Lewandoski *et al*., 1997), and genotyping was performed as previously described. Efficiency of excision in the ZP3-Cre line was tested by the *ZAP*^*CAG-Bgeo/ALPP*^ reporter.

α-lipoic acid (ALA), resveratrol (Sigma-Aldrich, Oakville, Ontario, Canada), and CoQ10 (Sigma-Aldrich or Advanced Orthomolecular Research Inc., Calgary, Alberta, Canada) were dissolved in sesame oil. Nine-month-old mice were injected subcutaneously with doses of ALA (33 mg kg^−1^), resveratrol (10 mg kg^−1^), CoQ10 (22 mg kg^−1^), or placebo (sesame oil) three times a week for a period of at least 12 weeks as shorter treatment did not show beneficial effects in preliminary breeding trials. Treatment for 12–13 weeks was used for the analysis of various outcomes in ovulated oocytes. For rescue experiments in the *Pdss2* model, mothers during pregnancy and their offspring after weaning received CoQ10 (LiQsorb, Tishcon, Westbuty, NY, USA) in drinking water (0.4 mg mL^−1^) (Saiki *et al*., 2008).

For breeding performance, dams were set with young male studs of proven fertility and checked daily for signs of pregnancy and delivery. Dams were pretreated for 12 weeks and set to breed while still being treated for one more month period (e.g., final treatment up to 16th week). Treatment (injections) was stopped when female was pregnant (as determined by gain of weight ∼ day 10.5 postmating). All together aged females were maintained with males for 4 weeks and 3 more weeks afterward, while young *Pdss2* females were monitored for at least 4 months. Twenty-one aged ICR females were used, although only 15 produced a single litter during this period in the control group. Twelve females were used for CoQ10 treatment, from which 11 produced a single litter during the breeding trial.

### Ovulation induction, oocyte collection, and assessment of ovarian reserve

Mice were superovulated with pregnant mare serum gonadotropin (ProSpec, Rehovot, Israel) and 48 h later with human chorionic gonadotropin (hCG) (Sigma-Aldrich) by intraperitoneal injection. For young females, 5 IU of both gonadotropins was administered, while old females received 10 IU. Mice were sacrificed by cervical dislocation and oviducts were removed. The oocytes were retrieved in modified human tubal fluid (LifeGlobal, Guilford, CT, USA) supplemented with 0.1% BSA (Sigma-Aldrich) and denuded of cumulus cells using hyaluronidase (Sigma-Aldrich). Murine germinal vesicle (GV) stage oocytes were collected 48 h after PMSG priming. Human immature oocytes (GV) not used for insemination were obtained from women aged 27 to 45 years who were undergoing IVF treatment. The study was approved by Mount Sinai Hospital (MSH REB Number: 05-0044-E), consent was obtained from all subjects, and experiments conformed to the principles of Declaration of Helsinki.

Murine ovaries were fixed in Dietrich’s fixative, embedded, serially sectioned (fully), and stained, and the number of healthy follicles at various stages of development was determined by systematic counts on every tenth section, summarized, and multiplied by a factor of 10. For excision efficiency, ovaries of *ZAP*^*ZP3-Cre+*^ females were collected at 2 months of age, and frozen sections were stained for alkaline phosphatase activity as previously described (Lobe *et al*., 1999). The number of excised oocytes (e.g., blue) was determined by counting 5 random ovarian sections from three females.

### Metabolic Assays, Mitochondrial labeling, and spindle scoring

Microanalytical metabolic assays for citrate, malate, and fumarate levels were performed as previously described (Chi *et al*., 2002), and for the ATP assay, Cell Titer GLO (Promega Madison, WI, USA) was used using ATP as standard. Ovulated oocytes were stained with MitoTracker Red, ROS – Mitosox (Invitrogen, USA), DePsifer (Trevigen, Gaithersburg, MD, USA), and autofluorescence for FAD (FITC) were performed as previously described (Fernandes *et al*., 2012). For oxygen consumption (MitoXpress Intra; Luxcel, Cork, Ireland), zona pellucida was removed with acid Tyrode’s solution, and oocytes were cultured for minimum of 5 h in the presence of probe at concentration recommended by a manufacturer and subsequently imaged on a spinning disk confocal microscope using appropriate filters. Ten optical sections from each sample were taken 1 min apart from each oocyte and were analyzed by Volocity Image Analysis Software, PerkinElmer Inc., Waltham, MA, USA. Final data are shown as ratio of these values. ATP and oxygen consumption assays were validated by treatment of oocytes with 8 μm antimycin for 30 min (Fig. S5).

Oocytes were scored for appearance of the spindle structure and arrangement of the chromosomes. Normal oocyte contained a barrel-shaped spindle with condensed chromosomes positioned centrally on the equator of the spindle. Abnormal spindle morphology included a reduction in the number of microtubules or the size of the spindle and detachment of the spindle from the chromosomes. Dispersion or misalignment of chromosomes from chromosomal plate was also defined as abnormal.

### Immunostaining

Ovarian sections fixed in 10% neutral buffered formalin were embedded in paraffin, sectioned, and rehydrated. Antigen retrieval with citrate buffer was used prior to exposure to antibody PDSS2 (1:40) followed by ABC Vectastain Kit (Dako, Glostrup, Denmark). Sections from *Pdss*^*fl/fl ZP3-Cre*+^ females were used as a negative control. Isolated oocytes at the GV stage were fixed in PHEM fixative, and indirect immunocytochemistry of the oocytes was performed as previously described (Fernandes *et al*., 2012). Primary antibodies included mouse antitubulin (1:500; Invitrogen) and rabbit anti-CoQ6 or PDSS2 (1:100; 1:200, respectively; Proteintech Group Inc, Chicago, IL, USA), followed by incubation with donkey anti-mouse Alexa 488 or donkey anti-rabbit Alexa 594 (Invitrogen). DNA was counterstained with 4′,6-diamidino-2-phenylindole (DAPI) and imaged on confocal microscope. Values obtained from samples without primary antibody were subtracted from the experimental signals, and the data were shown as the sum of intensity expressed as random fluorescence units (RFUs).

### Quantitative RT–PCR

Following oocyte retrieval, three oocytes were transferred into guanidinium isothiocyanate (GITC) solution, and cDNA for real-time PCR analysis was prepared as described (Perumalsamy *et al*., 2010). The sequences for forward and reverse primers are listed in Table S2. All gene expression experiments utilized the SYBR green PCR mix using LiteCycler (Roche, Mississauga, ON, Canada). qRT–PCR conditions were as follows: 95 °C for 10 min and then 40 cycles of 95 °C for 30 s, 60 °C for 30 s, and 72 °C for 30 s. Comparisons of expression levels were determined by delta CT method normalized to β*-actin*.

### Western blot

Ovarian protein lysates were prepared in 1% SDS-RIPA buffer containing a complete protease inhibitor cocktail (Roche) and protein concentrations determined using the BCA protein assay. Protein samples were resolved through 12% acrylamide gels and transferred to PVDF membranes. After blocking, the blots were probed with rabbit anti-CoQ6 or anti-PDSS2 (1:400 and 1:600, respectively; Proteintech Group Inc) followed by hybridization with goat anti-actin (Santa Cruz Biotechnology, Dallas, Texas, USA) as a loading control. The blots were then washed and incubated with appropriate HRP-conjugated secondary antibodies and ECL Plus.

### Ubiquinone extraction and measurement

For determination of ovarian ubiquinone (UQ) concentrations, whole ovaries and livers were homogenized in 0.5 mL of homogenization buffer (0.25 m sucrose, 10 mm Hepes buffer pH 7.4, 1 mm EDTA) with ten passes of the Teflon pestle homogenizer (Wheaton Overhead Stirrer, Wheaton Instruments, Millville, NJ, USA). After the total volume was made up to 500 μL with the homogenization buffer, 5 μL of the homogenates was used to determine their total protein content using the Bradford Reagent (Bio-Rad Mississauga, ON, Canada). To extract UQ, whole ovarian homogenates were mixed with an equal volume of hexane/ethanol for 10 min by vortexing. After centrifugation at 9000 *g* for 10 min, the hexane layer was collected and evaporated to dryness using a vacuum centrifuge (Eppendorf, Mississauga, ON, Canada). The quinone residue was then dissolved in 100% ethanol and analyzed by HPLC with UV detection at 275 nm (Beckman System Gold, Beckman Coulter Inc, Brea, CA, USA). A reverse phase C18 column (25.0 × 0.46 cm, 5 μm, Hichrom, Berkshire, UK) was used with an isocratic elution at a flow rate of 1.8 mL min^−1^. The mobile phase was methanol/ethanol (70:30 v/v). The concentrations of ubiquinones were estimated by comparison of the peak area with those of standard solutions of known concentration. Finally, quinone amount was normalized to protein content in the samples.

### Statistical analysis

All results are given as mean ± SEM. All statistical tests were performed with SigmaPlot 11 (Systat Software Inc., San Jose, CA, USA). Analyses were performed either with a one-way anova followed by Tukey’s or Dunn’s post-test, Mann–Whitney rank sum test, with the student’s t-test or chi-squared analysis, where appropriate. Results were considered statistically significant if *P* < 0.05.

## Supporting Information

**Fig. S1**. (A) Ovulation rate of 12 months old mice from control vehicle (*n* = 9), CoQ10 (*n* = 8), ALA (*n* = 7) and Resveratrol (*n* = 9) treated females. The CoQ10-treated group was the only one to show significant increase in ovulation rate compared to control. (B) Ovarian follicle reserve in ICR retired breeders at 9 month of age at the start of treatment and after 12 month of age (data for 12 months is the same as those in Fig. ). **Fig. S2. (**A) Level of gene expression (mRNA) in GV oocytes from young, old and old-CoQ10 treated mice which did not show significant change with age and/or with treatment. Values represent ratio of β*-actin* to target gene levels. (B) Fumarate levels were not significantly reduced by age, but were upregulated by CoQ10 treatment. **Fig. S3.** Validation of ZP3Cre activity in oocytes. (A) Ovaries from *ZAP*^*CAG-Bgeo/ALPP ZP3-Cre+*^ mice. Cre activity is restricted to oocytes and is initiated already at primordial follicle stage (arrows). (B) PDSS2 protein levels were significantly reduced in growing GV oocytes of 3 weeks old *Pdss2 *^*fl/fl Cre+*^ (*n* = 14) compared to *Pdss2 *^*fl/fl Cre−*^ (*n* = 12), indicating excision efficiency. (C) Antral follicles with fully grown oocyte present absence of Pdss2 activity in *Pdss2 *^*fl/fl Cre+*^ mice (arrowheads), while immunoreactivity is still observed in the cumulus cells surrounding the oocyte (arrows). (D) Breeding performance of young *fl/fl* and *fl/fl Cre+* mice during 4 months breeding trial. **Fig. S4.** Supplementation of with CoQ10 (LiQsorb) has no effect on (A) breeding (e.g. litter size), (B) ovulation rates, (C) ovarian reserve, (D) mitochondrial respiration (Mitotracker Red intensity or ROS production) and E) ovulation of chromosomally abnormal oocytes in young (up to 3–4 month of age) Pdss2 *fl/fl* (WT) females and (F) ATP level. However, similar to administration of subcutaneous CoQ10, LiQsorb did improve oocyte ATP level in 12 month old treated Pdss2 *fl/fl* females (G). **Fig. S5.** Validation of ATP and Oxygen consumption assay. Ovulated oocytes were collected and cultured in HTF medium with or without antimycin for 30 min followed by measurement of ATP (nm) (A) or oxygen consumption based on imaging of MitoXpress Intra (B) expressed as ratio of probe fluorescence taken in a 1 min interval. Data shown are mean ± SEM *n* = 16 oocytes for ATP experiment and *n* = 10 for oxygen consumption experiment. Both assays show significant reduction of signal (*P* < 0.001) after inhibiting activity of Complex III.

**Table S1.** Level of CoQ9 and CoQ10 (ng mg^−1^ of protein) after supplementation in various tissues. **Table S2.** List of primers used for gene expression studies.

** **
