# Supplementary figures and images for: Coenzyme Q10 restores oocyte mitochondrial function and fertility during reproductive aging
Source: Aging Cell. 2015 Jun 26;14(5):887–95. doi: 10.1111/acel.12368 (PMC4568976; doi:10.1111/acel.12368)

A

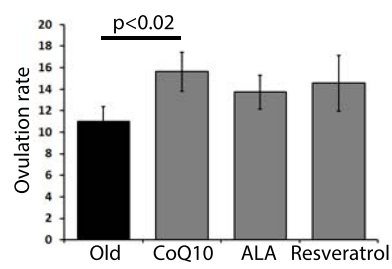

B

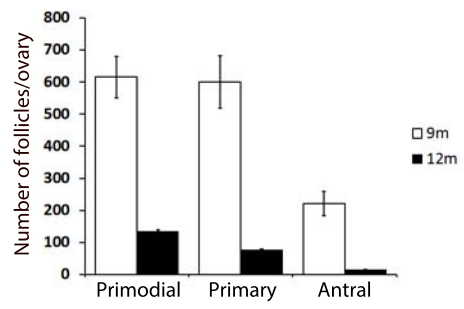

A

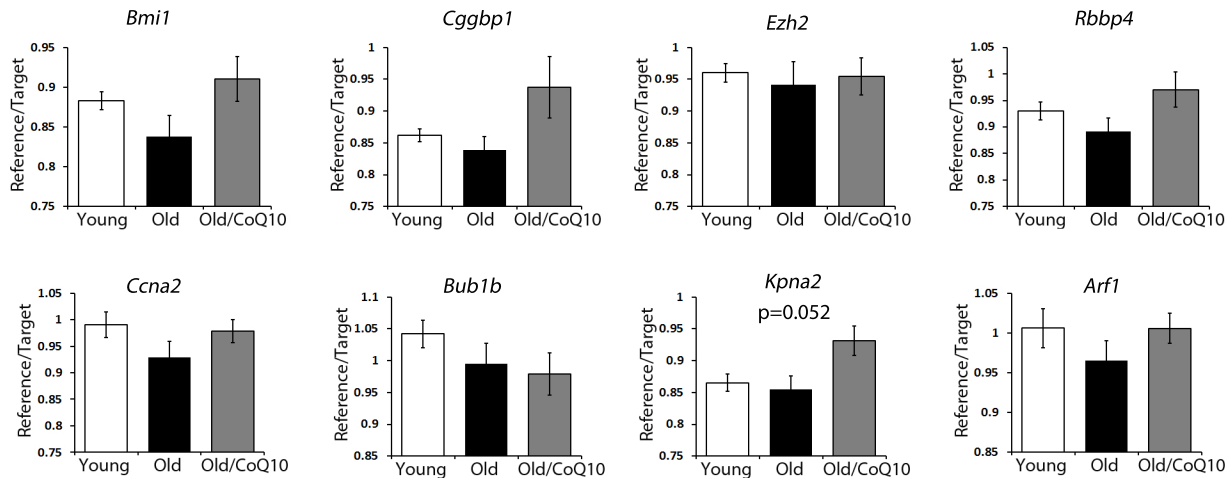

B

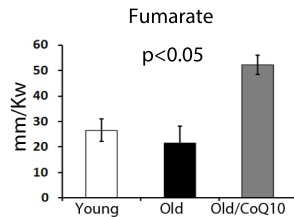

A

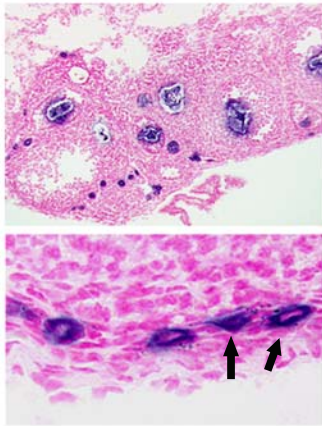

B

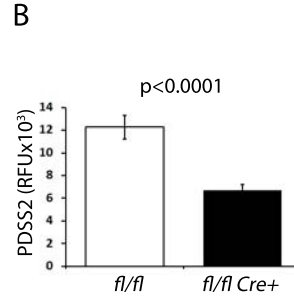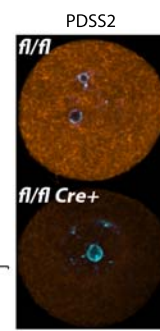

C

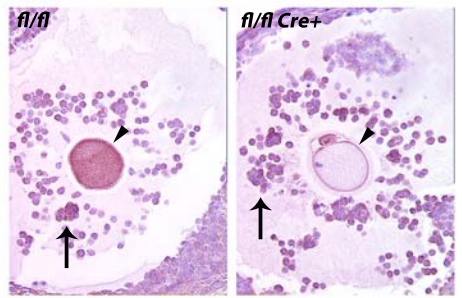

D

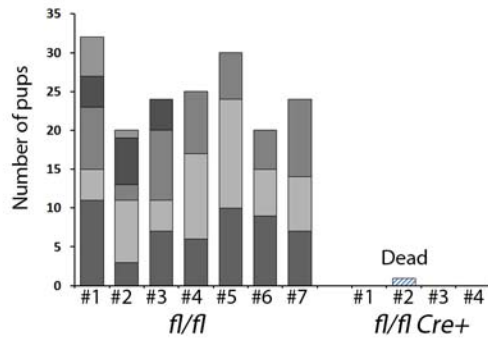



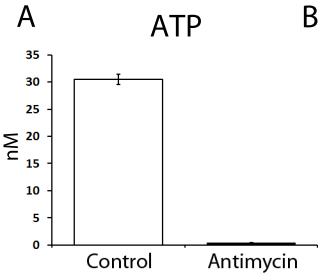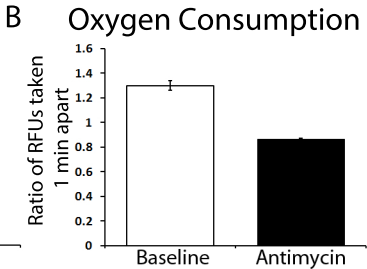

Supplement: Supplementary file 1 [file acel0014-0887-sd1.pdf]
