# Supplementary material for: Coenzyme Q10 restores oocyte mitochondrial function and fertility during reproductive aging
Source: Aging Cell. 2015 Jun 26;14(5):887–95. doi: 10.1111/acel.12368 (PMC4568976; doi:10.1111/acel.12368)
Supplement: Supplementary file 2 [file acel0014-0887-sd2.docx]

**Table S1.**

Level of CoQ9 and CoQ10 (ng/mg of protein) after supplementation in various tissues. Data shown are Mean ± SEM (n = 3-5 mice per group). u.d., undetectable; n.d., not determined. ** *p*<0.01 versus vehicle treatment (unpaired *t* test).

| **tissue**  treatment | Liver | | Kidney | | Ovary | |
| --- | --- | --- | --- | --- | --- | --- |
|  | CoQ9 | CoQ10 | CoQ9 | CoQ10 | CoQ9 | CoQ10 |
| Vehicle | 337.5  ± 21.4 | u.d. | 2601.9  ± 106.1 | 217.2  ± 13.3 | 1590.9  ± 618.2 | 67.2  ± 1.0 |
| CoQ10  Subcutaneous | 401.0  ± 35.2 | 31.4  ± 2.8 | n.d. | n.d. | 1613.3  ± 117.8 | 227.2  ± 34.3** |
| LiQsorb  Drinking | 378.1  ± 10.8 | 171.3  ± 37.2 | 2431.1  ± 307.1 | 238.8  ± 43.1 | 1390.9  ± 197.8 | 766.9  ± 144.6** |

**Table S2. List of primers used for gene expression studies.**

| Gene | Sequence |
| --- | --- |
| *Β-actin* | (F) 5’-CCACAGCTGAGAGGGAAATC-3’(R) 5’-AAGGAAGGCTGGAAAAGAGC-3’ |
| *Adck3* | (F) 5’-GACTTCGGTACCCAGAGCAC-3’ (R) 5’-TTCCTCGAACATGGCCTTAC-3’ |
| *Arf1* | (F) 5’-GAGGGTCGGTCCATTACAGA-3’ (R) 5’-GTCGTCTTTCGATGGCAAAT-3’ |
| *Bmi1* | (F) 5’-TTTTGGGAACCCTGTAGTGG-3’ (R) 5’-TTCACCAAAATTGGCAAACA-3’ |
| *Bub1b* | (F) 5’-ATGCCAGTGACAAGTCCACA-3’ (R) 5’-GCTGAGTGAGCAAAGCTTCC-3’ |
| *Ccna2* | (F) 5’-CTTTCCTTTTTCCCCAGAGG-3’ (R) 5’-AAAATGGGCAAACAAAGCAG-3’ |
| *Cggbp1* | (F) 5' -TCAACTGCCATTGAGTGAGG-3' (R) 5' -TCAGCAGTCTGGGAGTAGGG-3' |
| *COQ2* | (F) 5’-AAGAACAGCCAATCGTCCAA-3’ (R) 5’-CTGTCCCCCAAGAAAAACAA-3’ |
| *Coq2* | (F) 5’-ATCCACAGAGCCGAGGACT-3’ (R) 5’-TCAGCTCGTCTGCTCACTTC-3’ |
| *Coq4* | (F) 5’-CTGACTTCCGTCCCCAAGA-3’ (R) 5’-ACATCTCCCCAAACCATCAC-3’ |
| *COQ6* | (F) 5’-CTAAGCAGTTGGAGGCTGTGT-3’ (R) 5’-ATTTGGTCTGGAAGGTGCTG-3’ |
| *Coq6* | (F) 5’-GGCTGCTACTGACCTGTTGA-3’ (R) 5’-AGGAGACACTGCGTTCGTG-3’ |
| *COQ9* | (F) 5’-CGAGGAGGAGGAGGACTATGA-3’ (R) 5’-CCTTCTGCAATCGCCTCT-3’ |
| *Coq9* | (F) 5’-CTGGGAGGAGCAGCAGTAGA-3’ (R) 5’-TTGTGATTTCCAGCCTTGTG-3’ |
| *Ezh2* | (F) 5’-TGAAGTATGTGGGCATCGAA-3’ (R) 5’-CACTTTGCAGCTGGTGAGAA-3’ |
| *Hook1* | (F) 5’-TTGTAGCCACGTTTCCATCA-3’ (R) 5’-TGTCTCCCTTGTGTCTGCTG-3’ |
| *Kpna2* | (F) 5' -GAAGAGTGTGGAGGCTTGGA-3' (R) 5' -TTTCTGGCACCACATTTTGA-3' |
| *Msh3* | (F) 5' -TTTCATACCAAAGCGGGTTC-3' (R) 5' -CCTTTCCTTCCTGTGCTCTG-3' |
| *Ndufs3* | (F) 5' -TTATGGCTTCGAGGGACATC-3' (R) 5'-ATTCTTGTGCCAGCTCCACT-3' |
| *Nek2* | (F) 5' -TAGCATVTGGGGTCTCTTGC-3' (R) 5' -AGATTGTCAGGCCCTTCCTT-3' |
| *PDSS1* | (F) 5’-CTTTGATGGGAAAGGGAAAG-3’ (R) 5’GCCATTAGCGCCACAATAA-3’ |
| *Pdss1* | (F) 5’-TTTTACCAGACTGTGCCTAATG-3’(R) 5’-CCGAAAAACTTTGCCAATAAA-3’ |
| *PDSS2* | (F) 5’-TTGGTGGTGCTCCTTATCTCT-3’ (R) 5’-CATGAGTAGATCCCACTGACC |
| *Pdss2* | (F) 5’-CAGGAGAGCTTTTGGTGATG-3’(R) 5’-TCCTTCTCGTTTTGTTTGTAGTG-3’ |
| *Rbbp4* | (F) 5’-ATGCTACAAGCCCAAACCAC-3’ (R) 5’-AGGAGGAAGGAAAAACCACA-3’ |
| *Sdha* | (F) 5' - GCGTATGTGCTGGCTAGCTT-3' (R) 5' -AAGCCAATCCCTCAGAGACA-3' |
| *Smarca2* | (F) 5' -AGAAACACATGCGTGCAGAC-3' (R) 5' -CCACCCACATCTTTTCCATC-3' |
| *Sod1* | (F) 5' -GAGACCTGGGCAATGTGACT -3' (R) 5' -GTTTACTGCGCAATCCCAAT-3' |
| *Tuba1a* | (F) 5' -TCGTATCCACTTCCCTCTGG-3' (R) 5' -ACTGGATGGTACGCTTGGTC-3' |
