# Supplementary material for: Coenzyme Q10 restores oocyte mitochondrial function and fertility during reproductive aging
Source: Aging Cell. 2015 Jun 26;14(5):887–95. doi: 10.1111/acel.12368 (PMC4568976; doi:10.1111/acel.12368)
Supplement: Supplementary file 3 [file acel0014-0887-sd3.docx]

**Supplementary Figures.**

**Figure S1**. A) Ovulation rate of 12 months old mice from control vehicle (n=9), CoQ10 (n=8), ALA (n=7) and Resveratrol (n=9) treated females. The CoQ10-treated group was the only one to show significant increase in ovulation rate compared to control. B) Ovarian follicle reserve in ICR retired breeders at 9 month of age at the start of treatment and after 12 month of age (data for 12 months is the same as those in Figure 1).

**Figure S2.** A) Level of gene expression (mRNA) in GV oocytes from young, old and old-CoQ10 treated mice which did not show significant change with age and/or with treatment. Values represent ratio of *β-actin* to target gene levels. B) Fumarate levels were not significantly reduced by age, but were upregulated by CoQ10 treatment.

**Figure S3.** Validation of ZP3Cre activity in oocytes. A) Ovaries from *ZAP^CAG-Bgeo/ALPP ZP3-Cre+^* mice. Cre activity is restricted to oocytes and is initiated already at primordial follicle stage (arrows). B) PDSS2 protein levels were significantly reduced in growing GV oocytes of 3 weeks old *Pdss2 ^fl/fl Cre+^* (n=14) compared to *Pdss2 ^fl/fl Cre-^* (n=12), indicating excision efficiency. C) Antral follicles with fully grown oocyte present absence of Pdss2 activity in *Pdss2 ^fl/fl Cre+^* mice (arrowheads), while immunoreactivity is still observed in the cumulus cells surrounding the oocyte (arrows). D) Breeding performance of young *fl/fl* and *fl/fl Cre+* mice during 4 months breeding trial.

**Figure S4**: Supplementation of with CoQ10 (LiQsorb) has no effect on A) breeding (e.g. litter size), B) ovulation rates, C) ovarian reserve, D) mitochondrial respiration (Mitotracker Red intensity or ROS production) and E) ovulation of chromosomally abnormal oocytes in young ( up to 3-4 month of age) Pdss2 *fl/fl* (WT) females and (F) ATP level. However, similar to administration of subcutaneous CoQ10, LiQsorb did improve oocyte ATP level in 12 month old treated Pdss2 *fl/fl* females (G).

**Figure S5.** Validation of ATP and Oxygen consumption assay. Ovulated oocytes were collected and cultured in HTF medium with or without antimycin for 30 minutes followed by measurement of ATP (nM) (A) or oxygen consumption based on imaging of MitoXpress Intra (B) expressed as ratio of probe fluorescence taken in a 1 min interval. Data shown are mean +/- SEM n=16 oocytes for ATP experiment and n=10 for oxygen consumption experiment. Both assays show significant reduction of signal (p<0.001) after inhibiting activity of Complex III.
